# Palliative treatment of endometrial cancer: what is the role of anastrozole in elderly women?

**DOI:** 10.1186/s12904-021-00719-0

**Published:** 2021-02-05

**Authors:** Barbara Gardella, Mattia Dominoni, Stefano Bogliolo, Chiara Cassani, Giulia Vittoria Carletti, Annalisa De Silvestri, Arsenio Spinillo

**Affiliations:** 1 Department of Obstetrics and Gynaecology, University of Pavia, Fondazione IRCCS Policlinico San Matteo, Viale Camillo Golgi 19, 27100 Pavia, Italy; 2 Gynecology Oncology Unit, European Institute of Oncology, Milan, Italy; 3 Service of Biometry and Statistics, IRCCS Fondazione Policlinico San Matteo, Pavia, Italy

**Keywords:** Anastrozole, Aromatase inhibitors, Endometrial cancer, Palliative therapy

## Abstract

**Background:**

Type I endometrial cancer is the most common gynaecological tumour in developed countries and its incidence is increasing also because of population aging. The aim of this work is to test the feasibility and safety of anastrozole as palliative treatment of endometrial cancer in elderly women ineligible for standard surgical treatment.

**Methods:**

Patients with histological diagnosis of type I endometrial cancer not suitable for surgical treatment were enrolled in this pilot study. Anastrozole was administered 1 mg daily orally after performing an accurate clinical and radiological staging. Validated questionnaire and self-reported outcomes were used to evaluate quality of life and compliance during the study period.

**Results:**

Eight patients with a mean age of 85 (range 80–88 years) were enrolled. All patients had endometrial cancer confined to the uterus, and none progression of disease was observed during the study period. A partial response to the therapy was reported in seven patients, while one patient had stable disease. Tumour symptoms improvement such as pain, vaginal bleeding and vaginal discomfort was reported. The endometrial thickness after twelve months has showed a reduction of 9.25 ± 4.77 mm. The average follow-up time was 18.25 months. Four women died for non oncological reasons, none death related to endometrial cancer was reported. Evaluation of symptoms showed a significant reduction of appetite loss and insomnia, while a significant increase of global health status and fatigue was reported.

**Conclusions:**

Our preliminary data suggested that the palliative use of anastrozole may be a suitable therapy for the proper management of early stages endometrial cancer in elderly women not suitable for surgical treatment with good compliance and tolerance.

**Trial registration:**

2013000840. Date of registration: 21/09/2013. URL: trials.sanmatteo.loc.

## Background

Endometrial cancer (EC) represents the most common gynaecologic malignant disease in Western Countries. Over 50,000 women each year were affected by EC in the United States, with a mortality rate of approximately 8,500 patients/year [1]. It seems to be more prevalent in developed countries and especially in geriatric women; in fact, the mean age at diagnosis is estimated to be around 68 years [2, 3]. Type 1 EC represents the most widespread type (80–90 % of all endometrial malignancy) and it is estrogen-dependent. Endometrioid adenocarcinoma, another name for type 1 EC, generally manifests as low grade with a very favorable prognosis (5-years survival rate, in fact, is considered to be approximately 83 %) [4]. The persistent estrogenic stimulation of endometrial tissue, due to endogenous and exogenous origin, represents the principal pathogenic mechanism for the development of this malignancy [5–9]. The most important risk factors include ovarian polycytosis, estrogenic hormonal therapy, early menarche, late menopause, tamoxifen therapy for breast cancer treatment, anovulatory cycles and obesity. In addition familial history of EC, Lynch's syndrome, hypertension, mellitus diabetes, and thyroid diseases are additional risk factors for the onset of EC [5–9]. In post-menopausal women, the main source of estrogen is derived from the peripheral aromatization of steroids by the aromatase enzyme. This enzyme is present in many human tissues such as the placenta, adipose tissue, skin, granulosa ovarian cells, skin fibroblasts, muscle, bone and brain. Furthermore, the activation and the transcription of this enzyme seems to be direct correlated to patient's age and body mass index and this phenomenon well explained the rationale of the increased risk of developing EC in obese and elderly women after menopause. Genic expression of aromatase enzyme is significantly increased in the endometrial cancer tissue and it represents an index of tumour cell proliferation and growth [10–14]. To our knowledge, based on current literature, there are no studies which focus on the use of aromatase inhibitors in the treatment of EC in elderly patients. As it is shown in our previous review of the literature, aromatase inhibitors may possibly be beneficial in the treatment of advanced/recurrent type EC1.

The primary purpose of this study is to analyze the possible role of anastrozole, in elderly patients with EC who are ineligible for surgical treatment, in terms of safety, efficacy, and clinical benefit. Secondarily, this study evaluates quality of life modifications, toxicity and tolerability of the drug in this high risk group of patients.

## Methods

Inclusion criteria were in women with primary EC Type 1, International Federation of Gynaecology and Obstetrics (FIGO) Stage IA-IB, age older than 75 years, not candidate for surgical treatment due to advanced age, general status or comorbidities. The exclusion criteria were: radiological suspicion of distant metastasis according to Response Evaluation Criteria in Solid Tumors (RECIST) v1.1, Eastern Cooperative Oncology Group (ECOG) performance status > 3, any recent or concomitant neoplastic diseases in the previous 5 years, and no previous endocrine therapy for cancer. The present trial was approved by the Ethical Committee of IRCCS Policlinico San Matteo Foundation, Pavia.

From 2015 all consecutive patients age older of 75 with diagnosis of type I EC referred to our hospital underwent full clinical examination and trans-vaginal ultrasound. At histological examination of endometrial biopsy performed during hysteroscopy status of estrogen receptor and/or progesterone receptor was systematically performed by immunohistochemistry. A full clinical history, blood count, coagulative study and serum biochemistry were performed in order to analyze hepatic and renal function. All of our patients underwent abdominal and chest computed tomography (CT) in order to exclude distant metastasis or local tumor invasion, and pelvic magnetic resonance (MR) in order to investigate the depth of myometrial invasion. All patients underwent anesthesiological evaluation in order to assess perioperative risk according to American Society of Anesthesiologists score classification. Patients selected to use anastrozole as palliative therapy instead of receiving laparosocopic/laparotomic hysterectomy and bilateral salpingo-oophorectomy were poor surgical candidate due to their health general status. Informed consent was obtained in order to protect the patients regarding the use of off-label therapy. The anastrozole schedule was 1 mg/daily orally in order to minimize the adverse effect of progestagens, such as deep-vein thrombosis. In accordance with the National Health Service Cancer Plan, the therapy was started within one month of EC diagnosis [14]. Patients were treated until disease progression, intolerable toxicity or death. Four months after starting the therapy, all patients underwent ultrasound examination and abdominal CT in order to evaluate the response to the therapy, than every 6 months. Tumor response was investigated by using RECIST criteria. Complete blood count, renal and liver functions were tested starting one month after initiation of therapy and every four months. In order to evaluate the quality of life in each of the patients who followed the anastrozole treatment, the authors administered the European Organization for Research and Treatment (EORTC)-Core Quality of Life Questionnaire(QLQC)30 questionnaire at different times: before starting the treatment, and every six months until discontinuation. The QLQ-C30 is based on five multi-item scales (physical, role, social, emotional and cognitive functioning) and nine single-items (pain, fatigue, financial status, appetite loss, nausea/vomiting, diarrhoea, constipation, sleep wellness and quality of life) [15]. The toxicity profile was established according to National Cancer Institute Common Terminology Criteria for Adverse Events v 4.0. During follow-up evaluations, the clinical response to the therapy was determined with the Word Health Organisation Handbook for Reporting Results of Cancer Treatment [16]. According to RECIST 1.1, physical examination, and ultrasound evaluation: complete response (CR) occurs when all pathologic lesions disappeared. Partial response (PR) when the total cancer lesion size showed a decrease in size by 50 % or more; progressive disease (PD) when the lesion increased by 25 % or more in total measured size; Not changed (NC) when it was not possible to establish the reduction of tumour size by 50 % or more. The present trial adheres to CONsolidated Standards of Reporting Trials guidelines. The compliance to the therapy is based on patient self-reported outcomes.

### Statistical analysis

Sample size consideration: 8 subjects represent a small sample size, but a repeated measures design with one group of 8 subjects measured 3 times achieves more than 80 % power if effect size is about 1.5.

Categorical variables were described as count data and percentage. Quantitative variables were described as mean and standard deviation if normally distributed (Shapiro-Wilks test); as median and interquartile range otherwise. Response to the therapy was described in terms of reduction of endometrial thickness, vaginal bleeding. For clinical variables only descriptive statistics are shown.

Subscales of the EORTC-QLQ30 questionnaire have been calculated following the manual instructions and is accordingly linearly transformed to obtain a 0-100 score. Change of subscale scores at 6 and 12 months with respect to baseline were evaluated by fitting population-averaged generalized equation models following the approach described in Liang and Zeger (1986) [17]. Each normally distributed subscale is assessed in a separate model with time as independent variable. The small sample size does not consent multivariable analysis.

All statistical tests were two-sided and a *p*-value < 0.05 was considered significant. Analysis were performed with the STATA v16 (StataCorp USA).

## Results

**Fig. 1:**
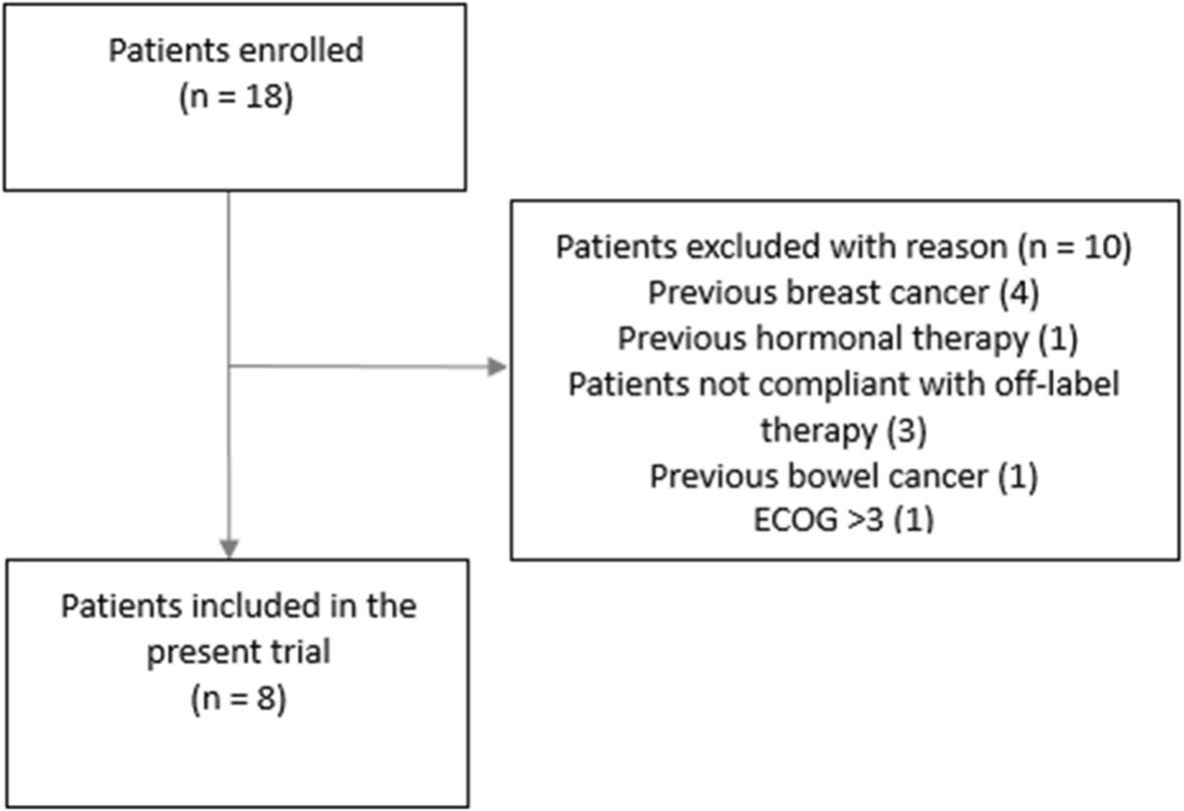
Flow of patients through the present trial

**Fig. 2:**
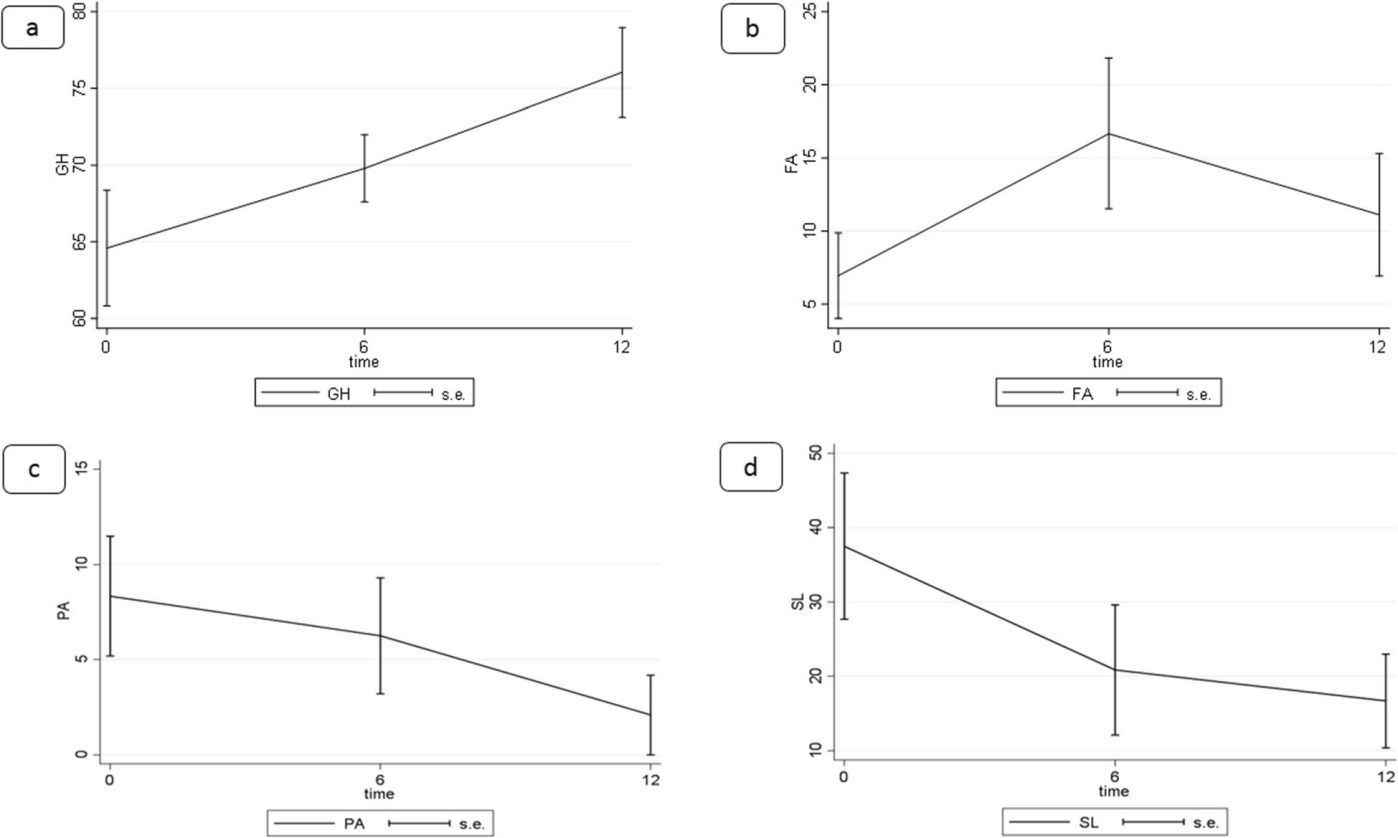
Graphic representation of items of EORCT-QLQ30 with p value statically significant. Legend: **a** GH: Global health status; **b** FA: Fatigue, **c**, PA: pain, **d** SL: Insomnia

Eight patients, according to inclusion criteria, were enrolled in the present study between 2015 and 2018, while ten women were excluded, as shown in the flow chart (Fig. 1). At the time of enrolment, the mean patient age was 85 ± 2.61 years (range 80–88 years). All patients had a diagnosis of endometrial cancer stage IA/IB, according to the FIGO system 2009. The histological and immunochemistry analyses demonstrated endometrial cancer type 1 in all patients (Table 1). Before starting the treatment, pelvic ultrasound, CT scan and Pelvic RM evaluated the lesion mapping: in all patients the tumour appeared confined to the uterus. The response to the therapy, counted such as any reduction in endometrial thickness, after twelve months was reported. The endometrial thickness had a mean of 10.74 ± 4.68 mm, with a reduction of 9.25 ± 4.77 mm (relative reduction rate of 44 %). The maximal endometrial thickness was 18 mm and the minimum was 5 mm (Table 2). Partial response was observed in seven patients (87.5 %, 7/8), with a reduction in self reported symptoms such as pain, vaginal bleeding, and vaginal discomfort for all patients. In one patient no endometrial thickness reduction was achieved during the follow up (12.5 %), and vaginal bleeding, pain and vaginal discomfort persisted, without any disease progression. The average follow-up evaluation was 18.5 ± 5.2 months for all patients. Four women died of reasons other than cancer during the study period: two patients died of heart failure following previous history of myocardial ischemia, one woman died after complicated femur fracture, another died after an accidental fall caused by pneumonia. No deaths related to endometrial cancer, disease progression/relapse or side effects from therapy were reported. Toxicity data were available for all patients included in the present trial. None of the patients reported any common side effects from the medication during the study period, such as vomiting, diarrhoea, alopecia, skin rashes and fever. Occasional nausea (4/8 patients), muscle/joint aches and bone pain (5/8 patients), and fatigue (5/8 patients) during the first month of the treatment were the only side effects reported. No grade 3–4 toxicity was highlighted. The therapeutic compliance was optimal thank to decrease in vaginal bleeding, subjective well-being and ease of oral administration and no patients stopped the anastrozole therapy due to adverse drug reactions. All eight patients filled out the EORCT-QLQ 30 questionnaire before, and at 6 and 12 months after starting therapy and results were summarized in Table 3. The global health status increased significantly 12 months after drug prescription (*p* = 0.002) (Fig. 2a). However, fatigue increased significantly at six months (*p* = 0.011) (Fig. 2b). We also reported a significant decrease in pain (*p* = 0.038) at 12 months compared to the baseline (Fig. 2c). Insomnia decreased significantly 6 and 12 months after the administration of anastrazole (*p* = 0.008 and *p* = 0.001, respectively) (Fig. 2d). No statistically significant changes from the baseline and follow-up evaluations were reported for other items of QLQ-C30.

**Table 1:** Demographic characteristics

Patients (age, years)	FIGO stage and grade	Comorbidity	ASA status	ER	PR	Symptoms T0 (^a^)	Symptoms T1 (^b^)
1 (85)	IA grade 1	atrial fibrosis, arterial hypertension, venous insufficiency of lower limbs and thrombophlebitis, hypertrophic cardiomyopathy	4	positive	positive	referred	not referred
2 (80)	IB grade 2	Parkison disease, ischemic stroke, arterial hypertension, bronchial asthma	4	positive	positive	referred	referred
3 (86)	IA grade 1	heart attack, high blood pressure, diabetes mellitus, obesity	3	positive	positive	referred	not referred
4 (82)	IB grade 1	chronic obstructive pulmonary disease, diabetes mellitus, arterial hypertension, thrombophlebitis lower limbs	3	positive	positive	referred	not referred
5 (88)	IA grade 2	arterial hypertension, previous post-traumatic pneumotorace, previous deep venous thrombosis	3	positive	positive	referred	not referred
6 (84)	IA grade 2	polymyalgia rheumatica, chronic pancreatitis, bilateral glaucoma, arterial hypertension, diabetes mellitus	3	positive	positive	referred	not referred
7 (87)	IA grade 1	thoracic aorta ectasia, arterial hypertension, diabetes mellitus	4	positive	positive	referred	not referred
8 (85)	IA grade 1	cerebral stroke, arterial hypertension, carotid atheromasia, obesity, diabetes mellitus	4	positive	positive	referred	not referred

(^a^): pain and vaginal bleeding before therapy

(^b^): pain and vaginal bleeding after therapy

**Table 2:** Endometrial assessments

	Mean ( ± SD)
Duration of anastrozole treatments ( months)	18.25 (± 5.20)
Endometrial thickness before therapy (mm)	20.25 ( ± 7.92 )
Endometrial thickness at follow-up 12 months (mm)	10.75 ( ± 4.68)
Maximum and Minimum endometrial thickness after therapy (mm)	18 and 5 mm
Reduction of endometrial thickness (mm)	9.25 (± 4.77)
Relative reduction of endometrial thickness (%)	44.16 (± 19.5)

**Table 3:** EORTC-QLQ30 items results at diagnosis and during follow-up evaluation

EORTC-QLQc30	Mean (±SD)			*P *	
	Baseline (0)	Six months (6)	Twelve months (12)	0 vs 6	0 vs 12
GH	64.58 (10.68)	69.79 (6.2)	76.04 (8.26)	0.156	***0.002***
PF	86.67 (11.27)	88.33 (9.92)	86.67 (10.69)	0.470	1
RF	97.92 (5.89)	100 (0)	93.75 (8.63)	0.401	0.093
EF	93.75 (7.39)	92.71 (5.34)	96.88 (6.2)	0.628	0.146
CF	100 (0)	95.83 (7.72)	97.92 (5.89)	0.131	0.450
SF	100 (0)	100 (0)	100 (0)		1
FA	6.94 (8.27)	16.67 (14.55)	11.11 (11.88)	***0.011***	0.276
NV	0 (0)	0 (0)	0 (0)		
PA	8.33 (8.91)	6.25 (8.63)	2.08 (5.89)	0.488	***0.038***
DY	12.5 (17.25)	16.67 (17.82)	12.5 (17.25)	0.190	1
SL	37.5 (27.82)	20.83 (24.8)	16.67 (17.82)	***0.008***	***0.001***
AP	0 (0)	0 (0)	0 (0)		
CO	8.33 (15.43)	0 (0)	8.33 (15.43)	0.121	1
DI	0 (0)	0 (0)	0 (0)		
FI	0 (0)	0 (0)	0 (0)		

*Legend*: *GH *Global health status, *PF *Physical functioning, *RF *Role functioning, *EF *Emotional functioning, *CF *Cognitive functioning, *SF *Social functioning, *FA *Fatigue, *NV *Nausea and vomiting, *PA *Pain, *DY *Dyspnea, *SL *Insomnia, *AP *Appetite loss, *CO *Constipation, *DI *Diarrhea, *FI *Financial difficulties, *SD *Standard Deviation

## Discussion

Our preliminary trial shows that anastrozole can be a valid palliative therapy in the treatment of EC in elderly women, which not only improves the quality of life, but also helps them to relieve the disease-related symptoms. Therefore the oral administration of anastrozole and low side effects improved compliance to the therapy in all of our patients. In fact, this treatment demonstrated a positive clinical response in 87.5 % of patients with an endometrial thickness reduction. The clinical response to the therapy was associated with global improvement and symptoms amelioration (especially vaginal bleeding and pain), also in cases of stable disease. In addition, Anastrozole therapy might prevent the onset of further recurrences and reduce the risk of endometrial hyperplasia.

Anastrozole is a third-generation non-steroidal aromatase inhibitor, which is able to bind to the heme group of cytocrome p-450 enzyme, so that the 99 % of the enzyme activity appears to be blocked [18, 19]. Previous study has shown that the use of aromatase inhibitors had a moderate clinical benefit in case of recurrent/advanced EC [19]. The review of Gao et al., suggested that the use of aromatase inhibitors appears to be a potential active therapy in endometrial cancer also in early stages [20]. Moreover Thangavelu et al. demonstrated that the markers of proliferation (KI-67 protein) and apoptosis (bcl2 protein) decrease in those patients with EC treated with anastrozole in neaodiuvant setting [21]. According to these biological and biochemical mechanisms, the administration of anastrozole for patients ineligible for surgery due to advanced age and/or comorbidities provides to be beneficial as a palliative treatment. Valenzano Menada et al. [22], demonstrated that endometrial thickness during anastrozole therapy decreased significantly also in breast cancer patients. The mean reduction reported by the authors was 4.5 mm in those patients with previous breast cancer treated with anastrozole and tamoxifen, while in our patients the reduction of endometrial thickness was 9.25 mm only with anastrozole. These difference can be related to a proliferative action of tamoxifen on endometrial tissue.

As the QLQ-C30 questionnaire reported, the global health increased significantly twelve months after beginning the therapy. The improvement of the quality of sleep at six and twelve months was reported by most of patients with psychological and physical benefits. Surprisingly we reported a significant increase of fatigue declared; we explained this result as related not to the side effects of anastrozole, but, more likely, to the advancing age of patients. Literature data were not concord about the significance of fatigue in elderly patients. Weims et al., reported that fatigue increased in patients underwent to palliative treatment comparing to adjuvant therapy, while Jing et al., showed that fatigue was common in elderly not cancer patients because of menopause and non oncologic gynecological diseases [23, 24]. Unchanged values reported in the other items of questionnaire are probably related to the early stage of the disease.

Currently, some other therapies are available for palliative treatment of EC, such as the use of progestagens, known since the 1950s, because they seem to have anti-oestrogenic activity by decreasing ER, by increasing oestrogen dehydrogenase enzyme, and by blocking the production of new receptors in endometrial tissue [25]. The use of aromatase inhibitors may be preferable thanks to better safety profile in pluripathological advanced aged patients. In fact, several sides effects have been reported by the use of progestagens, such as weight gain, hypertension, oedema, increases of blood sugar levels, sleep disturbance, tremor, bowel disturbance, and other more dangerous adverse effects such as thrombosis and pulmonary emboli [26, 27]. Nowadays progestin therapy by IUS system (Mirena) seems to be effective in the treatment of EC and in the decrease of malignant progression [28]. On the other side, oral therapy could avoid the discomfort caused by the IUS introduction in elderly women. Head-to-head studies in this setting are still lacking.

Even if our study enrolled a small sample of women, the prolonged follow-up and the quality of life data improve the significance of our results. Furthermore, larger and multicentric studies are necessary to confirm our data. In conclusion, minimally invasive surgery appears to be the best option in elderly women for the treatment of EC. Nevertheless, in those patients with reduced life expectancy, the primary goal of palliative therapy of EC is to reach an acceptable control of cancer symptoms, good tolerance of the therapy and an adequate quality of life: anastrozole could have all these characteristics.

## Data Availability

The datasets used and/or analyzed during the current study are available from the corresponding author on reasonable request.

## Abbreviations

EC: Endometrial Cancer
ER: Estrogen receptor
FDA: Food and Drug Administration
PR: Progesterone receptor
CT: Computed Tomography
MR: PMagnetic resonance
NHS: National Health Service
CR: Complete response
PR: Partial response
PD: Progression of disease
NC: Not changed
FIGO: International Federation of Gynaecology and Obstetrics
ECOG: Eastern Cooperative Oncology Group
EORTC-QLQC30: European Organization for Research and Treatment- Core Quality of Life Questionnaire C30
NCI CTCAE: National Cancer Institute (NCI) Common Terminology Criteria for Adverse Events
WHO: Word Health Organisation
CONSORT: CONsolidated Standards of Reporting Trials
